# Bullying among Brazilian adolescents: evidence from the National Survey of School Health, Brazil, 2015 and 2019*

**DOI:** 10.1590/1518-8345.6278.3678

**Published:** 2022-09-22

**Authors:** Deborah Carvalho Malta, Wanderlei Abadio de Oliveira, Elton Junio Sady Prates, Flávia Carvalho Malta de Mello, Cristiane dos Santos Moutinho, Marta Angelica Iossi Silva

**Affiliations:** 1 Universidade Federal de Minas Gerais, Escola de Enfermagem, Departamento de Enfermagem Materno-Infantil e Saúde Pública, Belo Horizonte, MG, Brasil.; 2 Bolsista do Conselho Nacional de Desenvolvimento Científico e Tecnológico (CNPq), Brasil.; 3 Pontifícia Universidade Católica, Campinas, SP, Brasil.; 4 Universidade Federal de Minas Gerais, Escola de Enfermagem, Belo Horizonte, MG, Brasil.; 5 Universidade de São Paulo, Escola de Enfermagem de Ribeirão Preto, Centro Colaborador da OPAS/OMS para o Desenvolvimento da Pesquisa em Enfermagem, Ribeirão Preto, SP, Brasil.; 6 Instituto Brasileiro de Geografia e Estatística, Diretoria de Pesquisas, Coordenação de População e Indicadores Sociais, Rio de Janeiro, RJ, Brasil.; 7 Universidade de São Paulo, Escola de Enfermagem de Ribeirão Preto, Centro Colaborador da OPAS/OMS para o Desenvolvimento da Pesquisa em Enfermagem, Departamento de Enfermagem Materno-Infantil e Saúde Pública, Ribeirão Preto, SP, Brasil.

**Keywords:** Bullying, Cyberbullying, Adolescent, School Violence, Schools, Health Surveys

## Abstract

**Objective:**

to estimate the prevalence rate of indicators related to bullying among Brazilian students aged 13 to 17 years and compare its occurrence between 2015 and 2019.

**Method:**

this is a descriptive cross-sectional study, with data from the National Survey of School Health, carried out in all Brazilian states. The prevalence rate and confidence intervals (95%CI) of the indicators were estimated in 2019. Student’s t test was used (p ≤ 0.01) to test the differences between editions.

**Results:**

the prevalence rate of bullying decreased from 20.4% (95%CI: 19.2 - 21.5) in 2015 to 12.0% (95%CI: 11.6 - 12.5) in 2019. The reasons cited for being bullied were similar in both editions: bodily appearance, facial appearance, and color/race. Prevalence rates were similar between states. The state of Tocantins presented the highest number of bully-victims; states of Mato Grosso and Amapá had the highest number of adolescents being involved in cyberbullying situations, and the state of Rio de Janeiro presented the highest number of bullies.

**Conclusion:**

there was a reduction by half in bullying and in the report on not being treated well among Brazilian adolescents; however, the prevalence rate of being bullied and cyberbullying are high in the country. Therefore, attention should be paid to policies to reduce and confront this issue on the national scene.

## Highlights

(1) Bullying is still significantly present among Brazilian students.

(2) The occurrence among boys from private schools stands out.

(3) Similar motivation in both editions: bodily and facial appearance, and color/race.

(4) Evidence for the implementation of actions and policies in the national territory.

(5) Contribution to the United Nations (UN) 2030 Agenda in the country.

## Introduction

School bullying is recognized as an emerging public health issue^1^ ^)-(^ ^3^. It is a type of violence characterized by systematic aggressions that are practiced intentionally^4^. Aggressive behavior marks the unequal power relationship between the peers involved in this type of situation^5^. As indicated in the scientific literature^1^ ^),(^ ^6^ ^)-(^ ^7^, school-age children’s and adolescents’ health and development are compromised when they are involved in bullying, which can occur both in the school context (traditional bullying) and in the cyber context, known as cyberbullying^8^ ^)-(^ ^9^.

In all countries around the world the prevalence rate of bullying in the school environment is high^10^ ^)-(^ ^11^. For example, in Jordan, the prevalence among bully-victims, bullies and bully-victims-bullies was of 16%^12^, while in Nigeria a prevalence of 50% student involvement in bullying has already been documented^13^. In Brazil, the National Survey of School Health (PeNSE) shows that in 2015 about 20% students reported bullying their peers, and 8% reported being bullied^5^. Globally, the prevalence rate of perpetration and victimization in the virtual environment also reaches variable levels according to the context. A systematic review showed that the prevalence rate of cyberbullying ranged from 6.0% to 46.3%, while the prevalence rate of victimization ranged from 13.99% to 57.5%^14^.

In this sense, mental health problems, decreased subjective well-being, greater emotional and behavioral issues, and lower levels of quality of life are aspects already associated with bullying^15^. A study carried out with students in 35 Western countries showed that being bullied decreases life satisfaction, constituting a mediating variable in relation to involvement in bullying situations^16^. It is known that life satisfaction is a component of psychosocial adjustment and can explain, to some extent, violence in schools, which should be perceived as components of the students’ community, safe and capable of promoting good social interactions^17^.

These aspects, related to the bullying magnitude and impact on the school-age children and adolescents’ health, disclose the relevance that the topic imposes on the field of health and nursing. Specifically nursing, as a social practice, has care as the essence and object of its work. This should be holistic and able to contemplate beyond the spaces naturally recognized as assistance or professional practice (health services, for example). In this context, nurses can develop health-related actions in schools and, considering the topic at hand, direct actions to reduce and prevent bullying, as well as mitigating its effects on students’ health. These actions should be guided by the perspective of health promotion and through clinical, educational, and administrative-management practices, considering bullying an object of care in the area^18^.

For this purpose, it is essential to broaden the understanding of the determinants involved in school bullying, given its high occurrence and health burden. In addition, with the advent of the COVID-19 pandemic, there is evidence of an increase in the prevalence rate of cyberbullying in several countries^19^ ^)-(^ ^22^; however, there is a lack of national and subnational studies on the subject. This gap was also made explicit in a recent literature review that aimed to understand the approach to primary health care in adolescence and related to cyberbullying, which revealed the challenges for nurses to be able to recognize this issue and propose care actions^23^. Therefore, PeNSE becomes an invaluable source of information on Brazilian adolescents’ health, making it possible to scale the scenario of student involvement in bullying and cyberbullying situations, subsidizing health promotion and prevention policies in the school context, in addition to be the baseline for other studies on the subject before the pandemic.

Thus, this study aimed to estimate the prevalence rate of indicators related to bullying among Brazilian students aged 13 to 17 years and compare its occurrence between 2015 and 2019.

## Method

### Study type

This is an epidemiological descriptive cross-sectional study, which adopted recommendations of the Strengthening the Reporting of Observational Studies in Epidemiology (STROBE)^24^. Data were collected from PeNSE, a national survey carried out by the Brazilian Institute of Geography and Statistics (IBGE) in partnership with the Ministry of Health, which provides information on 13- to-17-year-old students’ health^25^.

### Locus

PeNSE is conducted every three years in public and private schools in Brazil’s five main geographic regions including all Federation Units (UFs), Capital Cities, and the Federal District, and the data were collected between April and September 2015 and 2019.

### Population and sample definition

The study population is composed of Brazilian students aged between 13 and 17 years enrolled and attending 6^th^ to 9^th^ grades of Elementary School and 1^st^ to 3^rd^ grades of High School in public and private schools.

In 2015, IBGE used 2 samples: sample I composed of 9^th^-grade students, and sample II composed of students selected by age, from 13 to 17 years old, in 371 schools and 653 classes in the country’s five main geographic regions and the general total for Brazil^26^. All students from the selected classes present on the day of data collection were invited to participate in the research. Sample loss was approximately 2.4% considering enrolled students and non-respondents. More details on the sample can be found in another publication^26^.

In 2019, IBGE used a single sample composed of students aged 13 to 17 years, from 4242 public and private schools and 6612 classes, for the following geographic levels: Brazil, Major Regions, UFs, Capital Cities, and Federal District. Sample loss was 15.2% considering what was expected from the students and what was collected. More details are provided in another publication^27^.

The research sampling plan was defined as a sample of clusters in two stages, whose schools correspond to the selection first stage, and the groups of students enrolled to the second stage. The set of students from the selected classes formed the student sample. The selection of classes in each school in the sample was performed randomly with equal probabilities. All students were invited to answer the research questionnaire in the selected classes. Sample weights were used considering the weights of schools, classes, and students, and were adjusted based on the School Census data.

The PeNSE sample was dimensioned to estimate population parameters for adolescents aged 13 to 17 years, aiming to estimate a 0.5 (50%) proportion (or prevalence) *p* with a 4 % coefficient of variation (CV).

Besides, there are different samples between the two survey editions, explained in other publications^26^ ^)-(^ ^27^. However, the 2019 PeNSE sample is comparable to the 2015 PeNSE sample 2.

### Instruments used to collect data

Using smartphones, the students answered the structured and self-administered questionnaire, which was composed of information about socioeconomic status; family context; trying and using cigarettes, alcohol and other drugs; violence; safety; accidents, and their other living conditions.

All students were invited to answer the research questionnaire in the selected classes.

### Variables

**Figure 1:**
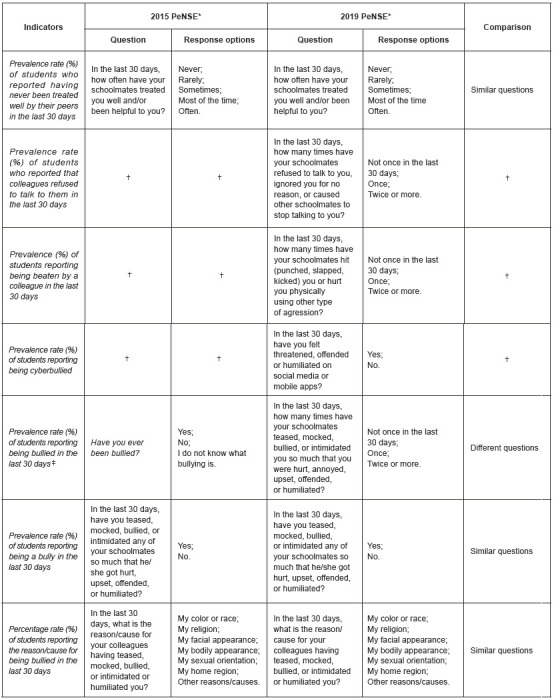
Description of indicators, questions and answer options regarding exposure to situations of violence by adolescent students. Brazil, 2015 and 2019 *PeNSE = National Survey of School Health; ^†^Means that this question was not present in the questionnaire in this survey edition; ^‡^Although this question was asked in both survey editions, the changes in the question and in the answer options made it impossible to compare this indicator

This study analyzed indicators referring to the module of situations at home and at school according to the research instrument and presented in Figure 1.

### Data collection

The data used are in the public domain and available in the Portuguese language on the IBGE website (https://www.ibge.gov.br).

### Data analysis

Initially, the prevalence rate and the respective confidence intervals (95%CI) were estimated according to sociodemographic variables (sex, age group, and type of school) and UFs and Regions in 2019. The 95%CI were used in the interpretation and comparison of estimates between different population groups, and a significant difference was considered when there was no 95%CI overlap^28^ ^)-(^ ^30^.

To test differences between the estimated proportions for 2015 and 2019, between similar indicators in the two editions, Student’s t test was used for independent samples, given that the samples in the two survey editions were selected independently. The estimated proportions and their respective variances were calculated considering the complex sample design; because of the multiple comparisons performed and the sample size in the two surveys, only the differences whose p-value was equal to or less than 0.01 were considered significant^31^ ^)-(^ ^32^.

Sampling structure and post-stratification weights were considered for all analyzes. Data organization and analysis were performed on Microsoft Office Excel software (Microsoft^©^, 2016).

### Ethical aspects

Both PeNSE editions comply with the Regulatory Guidelines and Norms for Research Involving Human Beings and were approved by the National Research Ethics Commission of the Ministry of Health (CONEP/MS), under opinions No. 1.006.467 of March 31^st^, 2015, and No. 3.249.268 of April 8^th^, 2019.

State and Municipal Secretariats of Education and the management team of the selected schools in each municipality were contacted before the study conduction. The students were informed about the research, their free participation and that they could withdraw if they did not feel comfortable answering the questions.

## Results

The 2015 PeNSE sample II consisted of 10,926 adolescents, 653 classes and 371 schools, with 50.3% boys and 49.7% girls; in 2019, the survey analyzed 4,242 schools, 6,612 classes and 125,123 students, with 49.3% boys and 50.7% girls.

It was observed that 7.2% (95%CI: 6.8 - 7.7) students aged 13 to 17 years reported having never been treated well by their peers, which was more frequent among boys (8.9%; 95%CI: 8.3 - 9.5) from 13 to 15 years (7.8%; 95%CI: 7.2 - 8.3) from public schools (8.1%; 95%CI: 7.6 - 8.5). The prevalence of being bullied twice or more in the last 30 days was reported by 23.0% (95%CI: 22.4 - 23.6) students and the percentages were higher among girls, 26.5% (95%CI: 25.6 - 27.2) aged 13 to 15 years (24.1; 95%CI: 23.4 - 24.8). No difference between students from private and public schools was found (Table 1).

**Table 1:** Prevalence and confidence interval of bullying indicators according to sociodemographic characteristics. National Survey of School Health (PeNSE). Brazil, 2019

Indicators	13 to 17 years of age					Age groups (in years)	
	Total	Sex		School			
		Male	Female	Public	Private	13 to 15	16 and 17
	% (95%CI)	% (95%CI)	% (95%CI)	% (95%CI)	% (95%CI)	% (95%CI)	% (95%CI)
Not being well treated	7.2 (6.8-7.7)	8.9 (8.3-9.5)	5.7 (5.2-6.1)	8.1 (7.6-8.5)	2.4 (2.1-2.6)	7.8 (7.2-8.3)	6.3 (5.7-6.9)
Being bullied	23.0 (22.4-23.6)	19.5 (18.8-20.2)	26.5 (25.6-27.3)	23.0 (22.4-23.7)	22.9 (22.3-23.5)	24.1 (23.4-24.8)	21.1 (20.2-21.9)
Being cyberbullied	13.2 (12.8-13.7)	10.2 (9.6-10.8)	16.2 (15.6-16.8)	13.5 (13.0-14.0)	11.8 (11.4-12.2)	13.2 (12.6-13.8)	13.3 (12.6-13.9)
Being beaten by schoolmates	6.5 (6.2-6.8)	8.2 (7.8-8.7)	4.9 (4.5-5.2)	6.2 (5.9-6.6)	8.3 (7.7-8.8)	7.8 (7.4-8.2)	4.2 (3.8-4.6)
Being a bully	12.0 (11.6-12.5)	14.6 (14.0-15.2)	9.5 (9.1-10.0)	11.8 (11.3-12.3)	13.5 (12.9-14.0)	12.2 (11.7-12.7)	11.7 (11.0-12.4)
Schoolmates did not talked to them	12.1 (11.7-12.6)	8.8 (8.3-9.4)	15.3 (14.7-16.0)	12.3 (11.8-12.8)	11.2 (10.8-11.7)	13.1 (12.5-13.6)	10.5 (9.8-11.1)

Regarding the prevalence of cyberbullying, 13.2% (95%CI: 12.8 - 13.7) students reported feeling threatened, offended and humiliated on social networks or cell phone applications in the 30 days prior to the survey, and the highest prevalence rate occurred among girls (16.2%; 95%CI: 15.6 - 16.8) from public schools (13.5%; 95%CI: 13.0 - 14.0) (Table 1).

The prevalence rates of bullying a colleague and being beaten by colleagues was 12.0% (95%CI: 11.6 - 12.5) and 6.5% (95%CI: 6.2 - 6.8), respectively. The report on bullying a colleague in some way was higher among boys (14.6%; 95%CI: 14.0 - 15.2) from private schools (13.5%; 95%CI: 12.9 - 14.0), with no differences between age groups. Being beaten by peers was more frequent among boys (8.2%; 95%CI: 7.8 - 8.7) from private school (8.3%; 95%CI: 7.7 - 8.8) and aged 13 to 15 years (7.8%; 95%CI: 7.4 - 8.2). It was found that 12.1% (95%CI: 11.7 - 12.6) students reported that peers refused to talk to them in the 30 days previous to the research, and it was higher in girls (15.3%; 95%CI: 14.7 - 16.0) from public school (12.3%; 95%CI: 11.8 - 12.8) and aged 13 to 15 years (13.1%; 95%CI: 12.5 - 13.6) (Table 1).

**Figure 2:**
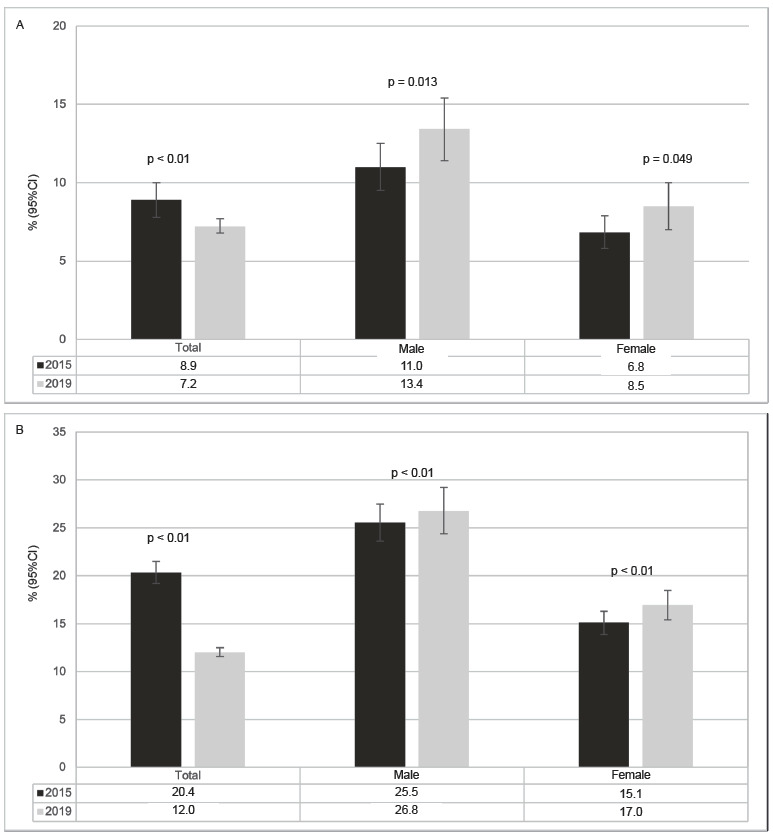
Prevalence and confidence interval of “not being treated well by schoolmates in the last 30 days” (A) and “being a bully in the last 30 days” (B) indicators, according to sex. National Survey of School Health (PeNSE). Brazil, 2015 and 2019

Figure 2 compares the only two similar indicators in both editions, and the prevalence rate of never having been treated well by colleagues was higher in 2015 (8.9%; 95%CI: 7.8 - 10.0) compared to 2019 (7.2%; 95%CI: 6.8 - 7.7). The prevalence rate of bullying a colleague in the last 30 days decreased from 20.4% (95%CI: 19.2 - 21.5) in 2015 to 12.0% (95%CI: 11.6 - 12.5) in 2019, both for girls and boys (Figure 2B).

**Figure 3:**
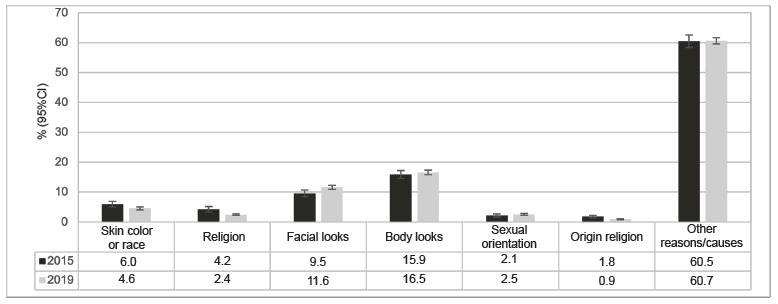
Percentage and confidence interval of the reasons or causes of being bullied among Brazilian adolescents. National Survey of School Health (PeNSE). Brazil, 2015 and 2019

The reasons for being bullied were similar in both editions, with emphasis on bodily appearance (15.9%; 95%CI: 14.6 - 17.2 in 2015 and 16.5%; 95%CI: 15.8 - 17.3 in 2019), facial appearance (9.5%; 95%CI: 8.5 - 10.6 in 2015 and 11.6%; 95%CI: 10.9 - 12.2 in 2019), and color/race (6.0%; 95%CI: 5.0 - 6.9 in 2015 and 4.6%; 95%CI: 4.0 - 5.1 in 2019) (Figure 3).

**Figure 4:**
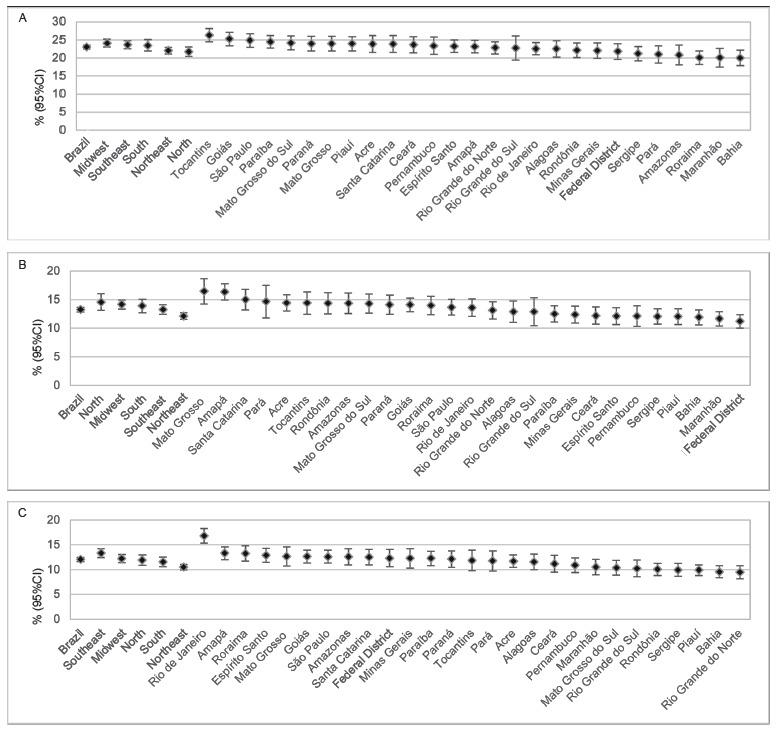
Prevalence and confidence interval of adolescents who report being bullied twice or more in the last 30 days (A), being cyberbullied twice or more in the last 30 days (B), and being a bully in the last 30 days prior to the research (C), according to Brazil, Regions and Federation Units. National School Health Survey (PeNSE). Brazil, 2019

Figure 4 presents “being bullied” and “being cyberbullied” indicators according to the Federative Units. The prevalence rate of being bullied twice or more in the last 30 days was similar in most states and regions. The highest frequency occurred among adolescents from Tocantins (26.3%; 95%CI: 24.4 - 28.2), and the lowest in Roraima (20.1%; 95%CI: 18.2 - 22.0) and Bahia (20.0%; 95%CI: 17.9 - 22.2) (Figure 4A). Regarding being cyberbullied twice or more in the last 30 days, the highest frequencies were observed in Mato Grosso (16.5%; 95%CI: 14.3 - 18.6) and Amapá (16.4%; 95CI% 15.0 - 17.8), and the lowest in the Federal District (11.2%; 95%CI: 10.0 - 12.4) (Figure 4B). Figure 4C shows the prevalence rate of adolescents who reported being a bully in the last 30 days in 2019, with Rio de Janeiro presenting the highest (16.8%; 95%CI: 15.4 - 18.3), and Rio Grande do North with the lowest (9.5%; 95%CI: 8.1 - 10.8) (Figure 4C).

## Discussion

The findings indicate that one out of four students reports being bullied, while the prevalence rate of being cyberbullied was approximately one out of nine adolescents in 2019. This prevalence decreased by half, as well as the report on not being treated well by schoolmates. The reasons given for being bullied were similar in both editions: bodily appearance, facial appearance, and color/race. It is noteworthy that more than half of the students did not attribute causes for the aggressions suffered. The prevalence rates were similar between the Federative Units. Tocantins presented the highest prevalence rate of students being bullied; Mato Grosso and Amapá had the highest rate of students being involved in situations of cyberbullying, and Rio de Janeiro had the highest number of students bullying someone.

The prevalence rate of students being bullied was high and similar to the results published by the United Nations Educational, Scientific and Cultural Organization (UNESCO), which, based on a global survey carried out on the prevalence rate of bullying in 68 regions worldwide identified even higher occurrences in Sub-Saharan Africa (48.2%), Middle East (41.1%), North Africa (42.7%), North America (31.7%), and South America (30.2%). The Caribbean (25%) and Central America (22.8%) presented rates similar to Brazil. Regarding the 71 countries analyzed, 13 presented increased prevalence rate; 35 showed a decrease in situations attributed to bullying, and 23 countries showed no change^10^.

Comparing PeNSE editions, there was a decrease in “never having been treated well by schoolmates” in 2019 compared to 2015. This indicator constitutes an important variable, as it denotes potential for the effective occurrence of bullying and other types of violence, in addition to impelling a conflict resolution model. In addition, this reduction can be attributed to greater awareness and approach to this issue at school in the country. However, it is known that bullying has important repercussions on students’ health and well-being, and evidence shows that adolescents who report not being treated well at school by their peers are about three times more likely to be bullied in relation to those who were well treated^33^.

In a study carried out with children and adolescents in situations of social vulnerability in order to analyze the sociometric status and its relationship with the profiles of participation in bullying, it was found that the aggressors’ high status can collaborate for them to use violence in the solution of conflicts or to obtain popularity among peers^34^. Furthermore, victims and witnesses of situations of violence at school can see and attribute this violent behavior as legitimate and appropriate for conflict resolution^35^.

The 2019 PeNSE also revealed that the report of being bullied was more frequent among girls aged 13 to 15 years who attend public schools and 13- to 15-year-old schoolchildren. Results from the Health Behavior in School-aged Children (HBSC) reflect similar findings, with a prevalence rate of 28.2% among girls and 30.1% among boys^10^. However, data found by the Global School-based Student Health Survey (GSHS), conducted with 317,869 students aged 12 to 17 years, showed that the global prevalence rate of bullying in the last 30 days prior to the survey was 30.5%, with almost a third of (33.0%) of male adolescents as victims, while in female adolescents the prevalence was lower (28.2%)^3^. Consequently, it is imperative to carry out further investigations into the variation in the prevalence rate of bullying among boys and girls in different countries, as it makes it possible to advance in the understanding of its social and cultural determinants.

These results reinforce the discussion about the difference between the sexes with regard to involvement in situations of violence between peers^3^ ^),(^ ^10^ ^)-(^ ^11^. In this sense, evidence of this nature can be explained considering that, as seen in Brazilian society, the hegemonic masculinity still reverberates in the school context, which imposes itself through aggressiveness and physical domination, establishing the dominant social representation of the man, determining in a strong manner the social roles to be played by boys. On the other hand, girls are more associated with forms of violence that are difficult to identify (verbal or symbolic, for example), but this may not necessarily mean that girls are less involved in bullying situations as aggressors^36^. This inequality is reflected in the bullying burden in the country: data from the Global Burden of Disease estimated that being bullied was responsible for approximately 118,000 disability-adjusted life years (DALYs) or 0.18% (95%CI: 0.045 - 0.42) of all DALYs in 2019 in Brazil, with this burden being higher among girls than among boys (75,293 or 0.25% (95%CI: 0.062 - 0.58) DALYs vs 42,888 or 0.12% (95%CI: 0.03 - 0.28) DALYs)^37^. Future studies are desirable, as they can help understand the differences in the way genders are related so that anti-bullying intervention measures are more effective.

The results of this study are also important when analyzing violence as one of the social determinants of health. In this sense, the report of the Lancet Commission on adolescents’ health and well-being^38^ ^)^ revealed that more than 50% of them grow up in countries with high levels of health issues among adolescents, including violence, evidencing the need to supervise and monitor its manifestations^3^. Specifically, the United Nations (UN) 2030 Agenda for Sustainable Development demands the systematic confrontation of bullying and, above all, reinforces the relevance of continuous monitoring of health indicators related to psychosocial aspects^39^ ^)-(^ ^41^.

On the other hand, cyberbullying was measured for the first time in a nationally representative sample of Brazilian adolescents, an aspect that is even more fundamental since an increase in this type of violence has already been observed during the global crisis caused by COVID-19^19^ ^)-(^ ^22^. Data on the difference between the sexes have also been evidenced when bullying occurred virtually^14^ ^),(^ ^42^ ^)-(^ ^43^. However, it is pointed out that bullying has not yet received the necessary attention, given its significance and the damage it can cause to the students involved - insomnia, depression, low school performance or low concentration, drug use, suicidal ideation and suicide, stress, and loneliness and anxiety, for example^44^. A systematic review of 66 studies showed that cyberbullying is associated with a higher risk of suicidal behavior and self-harm^45^. In this way, the results revealed by this study signal the importance that the topic will assume on the social and political agenda in the coming years, especially in the post-pandemic context, in view of the increase in screen time observed among adolescents^46^ ^)^ and, above all, the trend of increasing conflicts between peers after returning to school, due to the increase in mental suffering and anxiety generated by prolonged social distancing among young people.

Among the motives and causes of bullying, study participants mentioned bodily appearance, facial appearance, and skin color or race. These findings are not new and have already been documented in other PeNSE editions^47^ ^)-(^ ^48^. However, even with the dissemination of information about the issue, students still have difficulties in identifying the aggressors’ motivations. This aspect reinforces the importance of informative and formative strategies on school bullying. On the other hand, national and international studies seek to associate bullying with different variables to explain it, such as intrafamily violence; use and abuse of alcohol, tobacco and other drugs; age group, sexual orientation, bodily appearance; race; feeling of loneliness, and lack of friends^47^ ^-^ ^50^. Some of these variables are covered by PeNSE, but they were not mentioned by the participants when asked about the motivations for the aggressions suffered.

Although the study presents results that can be generalized to the experience of Brazilian students, its limitations have to be considered. The first limitation refers to the main outcome analyzed (bullying), as the questions were not comparable in the editions, making it impossible to assess changes over time. The change in the question between the previous editions and 2019 does not allow comparison with the tendency of being bullied among Brazilian adolescents. This was due to changes in the way in which questions were asked to students in the two editions. Therefore, it is suggested that the questionnaire be revised in order to allow an analysis of the temporal distribution of the event in question and, thus, recognize and measure its variation and identify the associated factors.

Furthermore, the cross-sectional design does not allow establishing causal links between the variables analyzed or exploring the mediating effects between the variables analyzed, in addition to being subject to information bias. Besides, this study reflects the situation of adolescents who are in school and not those who are out of it. In addition, the PeNSE module regarding bullying was not previously validated, which may result in some sort of bias. Studies of this nature are recommended to improve the research.

This study presents important strengths due to its magnitude, since it gathers data from the entire national territory and provides an overview of bullying indicators among Brazilian students. From previous PeNSE editions, it is reiterated that the Brazilian school context continues to be a space for (re)production of this type of violence, making it urgent to advance on the prevention and reduction of the multiple facets of bullying in this population, aiming at reducing the burden of associated mental disorders among adolescents^15^ ^),(^ ^40^.

## Conclusion

It was possible to observe that although there was a reduction in the prevalence rate of bullying among Brazilian students in 2019, this is still a serious issue on the national scene, especially among boys from private schools. Bodily appearance, facial appearance, and color/race are the main reasons for being bullied, denoting that the issue of physical appearance and tolerance of diversity should be considered in anti-bullying interventions. Cyberbullying has also revealed a troubling issue, particularly among girls and in public schools. Additional investigations are needed for the understanding of the individual and contextual determinants related to this type of violence among Brazilian students, especially after the COVID-19 pandemic.

The results summarized in this study also point to the importance of health care offered to children and adolescents involved in bullying and/or cyberbullying situations. In the meantime, the work in the field of nursing, especially in primary care, imposes itself as a challenge and an exercise to understand and transform collective health practices and intervention and prevention proposals, processes that require new knowledge that is capable of generate collective awareness and a commitment to the issues of inequality, exclusion, and discrimination to which many children and adolescents are exposed. In addition, multisectoral action is essential, especially among health, social service, and education actors, as they can collaborate to tracking cases and reducing the movement of naturalization or trivialization of violence between peers. This type of care is in line with the national guidelines for comprehensive health care for adolescents and young people in the promotion, protection, and recovery of health and with the national health promotion policy in conjunction with the *School Health* Program.
